# Incorporation of fragmented visuo-olfactory episodic memory into dreams and its association with memory performance

**DOI:** 10.1038/s41598-019-51497-y

**Published:** 2019-10-30

**Authors:** J. Plailly, M. Villalba, R. Vallat, A. Nicolas, P. Ruby

**Affiliations:** 1 Olfaction: from coding to memory Team, Lyon Neuroscience Research Center, CNRS UMR 5292 - INSERM U1028 - University Lyon1, Lyon, F-69366 France; 2 DYCOG Team, Lyon Neuroscience Research Center, CNRS UMR 5292 - INSERM U1028 - University Lyon1, Lyon, F-69366 France; 3 Centre Hospitalier Le Vinatier, Bron, F-69678 France

**Keywords:** Consciousness, Consolidation

## Abstract

The question of a possible link between dream content and memory consolidation remains open. After a comprehensive review of the literature, we present novel findings from an experiment testing whether the incorporation of recently learned stimuli into dream reports is associated with improved post-sleep memory performance. Thirty-two high dream recallers freely explored new visuo-olfactory episodes for 3 consecutive days. During the nights following each non-explicit encoding, participants wore a wrist actimeter, and woke up at 5am and their usual waking time to record their dreams (intensity of all oneiric sensory perception was assessed using scales). A total of 120 dreams were reported and elements related to the encoding phase were identified in 37 of them, either learning-related (mainly visual- and rarely olfactory-related elements), or experiment-related (lab- or experimenters-related elements). On the 4^th^ day, we found that participants with learning-related (n = 16) and participants with learning-related and/or experiment-related dreams (n = 21) had similar odor recognition and odor-evoked episodic memory with the other participants. However, they had significantly better visuo-spatial memory of the episodes in comparison to the other participants. Our results support the hypothesis that the learning phase is loosely incorporated into dreams and that this incorporation is associated with sleep related memory consolidation.

## Introduction

Sleep is essential for procedural and declarative memory consolidation^1–3^. Post-learning sleep is hypothesized to have a beneficial effect on memory through reactivation and reorganization of the memory trace at the cerebral level^4^ (and for reviews^5,6)^. Because the cognitive experience of dreaming during sleep^7,8^ regularly incorporates waking life elements^9–11^ (and for a review^12)^, the idea of a possible link between dream content and memory consolidation has rapidly emerged. A current hypothesis postulates that dream content reflects memory consolidation and predicts better post-sleep performance when a recent learning experience is incorporated into dreams^13–17^.

Only a few studies have experimentally addressed this issue and tested whether memory performance improved when participants reported learning-related dreams. An up-to-date and comprehensive review of these studies is presented in Table 1. Various kinds of tasks were used such as learning a story^18^, a foreign language^19^, meaningless sentences^20^, word-picture associations^21^, vertical inversion of the visual field with goggles^22^, the computer game “Doom”^23^, a virtual balancing motor task^24^, mirror tracing^25^ and navigating a virtual maze^26^. Inconsistent results were obtained: five studies reported better memory performance in participants who recalled learning-related dreams, one study reported inconclusive results, and six studies reported no relationship between memory performance and learning-related dreams. Even more puzzling, inconsistent results were reported with the same paradigm, in the same team, and across repeated studies^26–29^.

**Table 1:** Review of the experiments which investigated the link between learning-related dream reports and memory performance.

Ref	Memory	Task	N_t_	n_LRD_	Scoring	Dreams collection	Statistical paradigm	Res
(Fiss *et al*.^18^)	Explicit verbal	Story recall	6	?	Content checklist procedure	1 night in the lab with REM awakening	Correlation between inc of the story into dreams and memory performance	**YES**
(De Koninck *et al*.^19^)	Explicit verbal	French language learning	8	?	External-scoring: detection of French	Dream journal from pre-course to post-course	Correlation between learning efficiency and latency to first French inc into dreams	**YES**
(Wamsley *et al*.^26^)	Explicit visuo-spatial	virtual maze navigation task	50	4,8%	Self-scoring & experimenter validation	Awakenings in N1 and at the end of the nap	Group comparison	**YES**
(Schoch *et al*.^21^)	Explicit visuo-verbal	Word-picture association	22	?	External-scoring by 2 blind raters of congruent vs incongruent inc	1 night in the lab with multiple awakenings (REM & NREM)	Correlation between inc score and overnight memory retention	**YES** in NREM **NO** in REM
(Wamsley & Stickgold^27^)	Explicit visuo-spatial	virtual maze navigation task	17	8,47%	External-scoring by blind raters of direct explicit inc* of the maze	1 night in the lab with multiple awakenings in N1, 1 in N2 and 1 in N2 or REM sleep	- Group comparison (those with at least one direct inc vs those with no direct inc of the task into dreams) - ANCOVA	**YES** **NO**
(De Koninck *et al*.^22^)	Explicit visuo-spatial	Vertical inversion of the visual field with goggles	8	4,50%	External-scoring: incorporations of visual inversions	2 night in the laboratory with REM awakening	Better score on 2/3 of the tests of adaptation to the visual inversion for the participants with inc of the task into dreams (tendency)	**?**
(Cipolli *et al*.^20^)	Explicit verbal	Meaningless sentences (3 × 19 words)	12	12,100%	External scoring by 2 blind raters looking for loose associations with the stimuli in dreams resulting in 31/35 dream reports with inc of the task	Several awakenings in the lab after 5 min of REM sleep	ANOVA testing the retention rate for content words as a function of inc into dream reports + moment of recall + REM period	**NO**
(Pantoja *et al*.^23^)	Perceptuo-motor-spatial-emotional & higher level cognition	Computer game « Doom »	22	17,77%	Not specified	2 nights in the laboratory with REM awakenings in the 2^nd^ night	Correlation between the amount of game-related elements into dreams and performance gains (inverted U function)	**NO**
(Schredl & Erlacher^25^)	Procedural & visuo-spatial	Mirror tracing	20	1,5%	External-scoring: laboratory experiment and mirror tracing task references (binary score).	2 nights in the lab with REM awakenings in the second night (from the 2^nd^ REM period)	Correlation between reference to 1) the experiment, 2) the laboratory, and 3) the task into dreams and performance to the task	**NO**
(Stamm *et al*.^29^)	Explicit visuo-spatial	virtual maze navigation task	65	24,37%	External-scoring by blind raters of direct and indirect inc of the maze	1 night in the lab with multiple awakenings in N1, 1 in N2 and 1 in N2 or REM sleep	Group comparison (those with inc vs those with no inc of the task into dreams)	**NO**
(Wamsley *et al*.^28^)	Explicit visuo-spatial & procedural motor	virtual maze navigation task & motor sequence typing task	51	6,12%	External-scoring by blind raters of direct and indirect inc of the maze	1 night in the lab with multiple awakenings in N1, 1 in N2 and 1 in N2 or REM sleep	Group comparison (those with inc vs those with no inc of the task into dreams)	**NO**
(Nefjodov *et al*.^24^)	Procedural & visuo-spatial	Computer coordination and balance motor task	13	≥7,≥53%	Self- & external-scoring	1 night in the laboratory with REM awakenings (from the 2^nd^ REM period)	Correlation between reference to balance-related elements into dreams and task performance	**NO**

Ref, references; Memory, type of memory targeted by the task; Task, task performed before sleeping and which presence in dreams was scored; N_t_, total number of participants; n_LRD_, number and percentage of participants with learning-related dreams; Scoring, method for scoring whether dreams were learning-related or not; Dream collection, method used to collect dreams; Statistical paradigm, method used to test whether dreaming of the learning phase was associated with improved memory performance; Res, results i.e. response to the question “*Did the results show that the more the dreams are learning-related or the more learning-related dreams*, *the better the performance after sleep?*”; ?, unknown; inc, incorporation; N1, sleep stage N1; N2, sleep stage N2; * indirect incorporations excluded because not related to performance.

In addition to the studies’ discordant results, several limitations prevented these studies from conclusively answering the question of whether there is a link between the incorporation of a memory into dreams and memory consolidation. First, most of the studies had a low statistical power due to a small sample size in either the number of participants or in the number of dreams, which makes the interpretations and generalizations of these findings difficult. Second, the criteria used to score the incorporation of the task into dreams were often scarcely described although the pertinence of the test critically relied on the specificity with which the task could be recognized into dream reports. Third, most of these studies employed a serial awakening paradigm to collect multiple dream reports, which disrupts sleep and may thus also disrupt the sleep-related memory consolidation processes^1,30–33^ (only one study so far reported no declarative memory alterations the day after participants were awakened a few times per night to report dreams^21^). Fourth, while people mostly encode events implicitly in their daily lives, these studies mainly targeted explicit encoding memory tasks. Finally, not all the sensory modalities have been tested and therefore the possible interaction between dreaming-related memory consolidation and the sensory modalities involved remains unknown.

**Figure 1:**
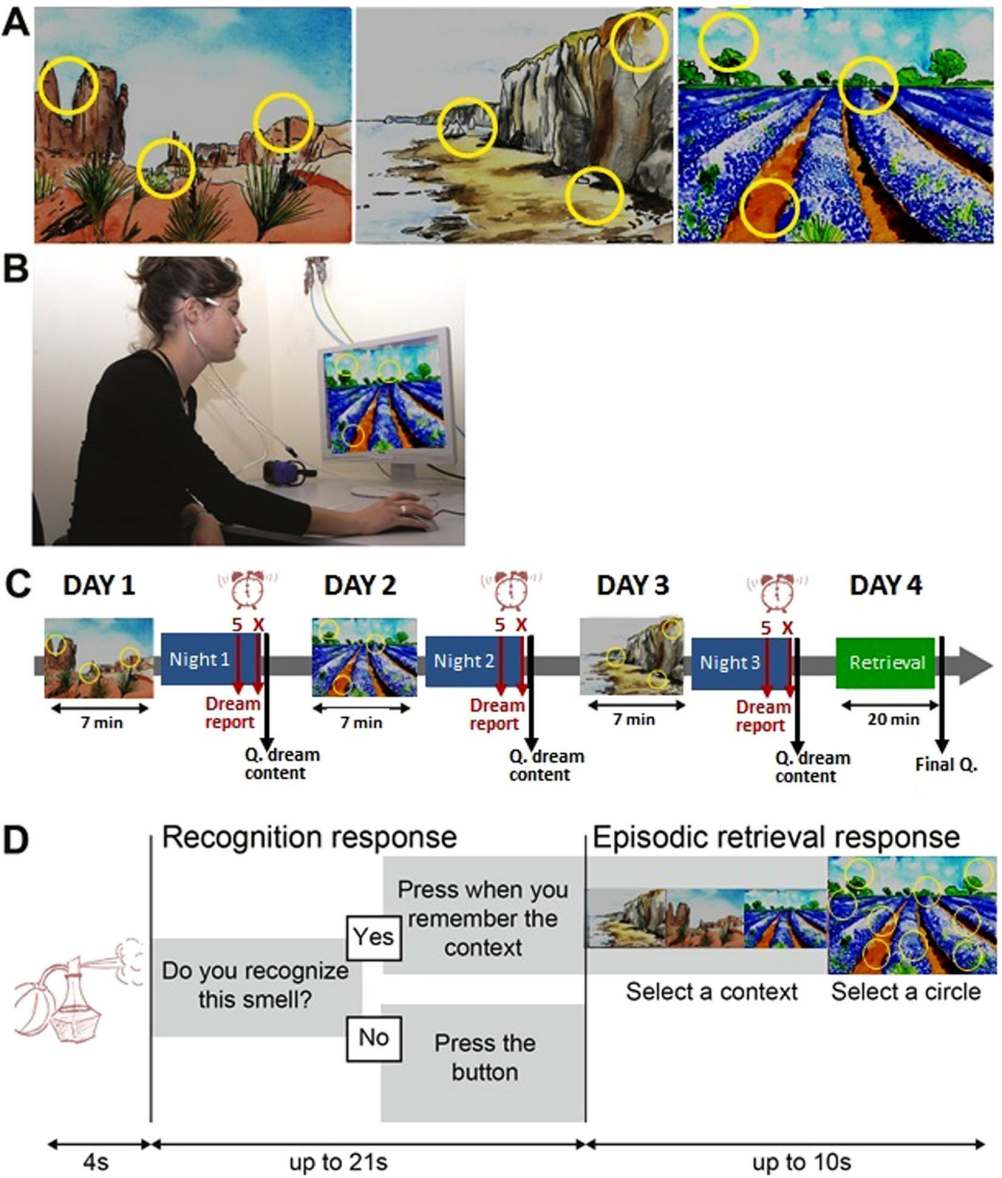
Experimental task. (**A**) Drawings of the 3 visuo-spatial contexts (drawings of the pictures used were made by Salomé Blain, the original pictures can be seen in Saive *et al*.^34,35^). The odors were presented when clicking in the yellow circles added on the pictures. (**B**) Photo showing the experimental setup with the breathing apparatus (copyright holder: Partick Minary, INSERM). (**C**) The temporal course of the encoding and retrieval sessions. During the encoding, the participants discovered one episode per day (during 7 min) over 3 days. During the night at home, they woke up at 5 am and at their usual waking time to record their dreams with a voice recorder (Dream report). In the morning they fill in a questionnaire about dream content (Q. dream content). The last day, they underwent a retrieval session and filled in a final questionnaire (Final Q.). (**D**) In the retrieval session, the memory of the episodes was tested using an odor-recognition task followed for the ‘Yes’ trials by an episodic memory retrieval task. The perfume bottle and the clock were drawn by the authors. All rights reserved, this image is not included under the Creative Commons licence for the article.

In an attempt to minimize the limitations listed above, the study was designed with the following characteristics. We chose a paradigm designed to investigate episodic memory^34,35^ of multisensory episodes (odors perceived at specific locations of a landscape picture, Fig. 1A). Each of the three episodes was experienced only once (i.e. on 3 successive days) by the participants for 7 minutes and without any explicit instruction to memorize it. The 32 participants (with a dream recall frequency superior to 4 mornings per week with a dream in mind^36–40^) slept at home with a wrist actimeter (to assess sleep duration) during the 3 nights following each encoding day and woke up at 5 am and at their usual waking time to report possible dreams with a voice recorder (orally reporting dreams is easier than writing). Their memory performance was tested on the fourth day (Fig. 1). To keep sleep disturbances at a minimum, dream reports were collected in a natural setting at home, without EEG recordings, and with only one intra-sleep awakening.

The first objective was to further test whether recalling a dream related to a recent experience is associated with improved memory performance, when memory encoding is not explicitly required and when odors are a part of the experience to be later recalled. The hypothesis proposing a link between memory consolidation during sleep and dream content^13–17^ has not been restricted to explicit memory, and some results have shown that olfactory memory is consolidated by sleep^41–43^. Better memory performance in participants with learning-related dream reports were thus expected. The postulate underlying this prediction is that the more one reports dreams with elements of the task or of the context of the task, the more one dreams of the task. The second objective was to discuss, at the theoretical level, the plausibility to test whether dreaming of a task is associated with improved memory performance.

## Materials and Methods

### Participants

Thirty two healthy persons [8 males; age: 21.94 ± 2.18 years (mean ± standard deviation)] participated in the study. Participants were selected based on their self-assessed (*a posteriori*) dream recall frequency (DRF; the threshold for selection was at least 4 mornings per week with a dream in mind^39^) which resulted in an average DRF of 4.89 ± 1.21 dream reports per week in the final sample. All participants reported normal senses of smell and vision, and no psychiatric, neurologic or sleep disorders (participants with a previously diagnosed disorder in any of these domains were excluded). All participants agreed to report dreams without censorship. The local ethical committee (Comité de Protection des Personnes Sud Est IV; ID RCB: 2015-A01595-44) approved this study on December 2015, 17^th^. The study was conducted in conformity with the Declaration of Helsinki. Participants provided written informed consent and received financial compensation.

### Stimuli and materials

#### Odorants

Eighteen odorants consisting of essential oils and single or mixtures of monomolecular chemical compounds, were selected *a priori* based on their distinctiveness and relatively low identifiability and familiarity (2-Heptanone, 9-decen-1-ol, basil, butanol, birch oil, carrot, cis-3-hexenyl salicylate, citronellol, dihydromyrcenol, ethyl acetyl acetate, linalyl acetate, methyl octane carbonate, musk, rosemarel, rose oxide, stemone, tobacco and tomato)^34,35^. The odorants were randomly subdivided into two sets (target, distractor) of nine odorants each for each participant.

The odorants were presented using a 20-channel computer-controlled olfactometer adapted from an olfactometer previously described by Sezille *et al*.^44^. Briefly, this odor diffusion system was developed to synchronize odorous stimuli with breathing. Undiluted odorants were contained in a 10-ml U-shaped Pyrex® tube (VS Technologies, France) filled with odorized microporous substances. Odorized airflows and air carrier were sent to and mixed in a homemade mixing head made of polytetrafluoroethylene and connected to the nostrils. The participant’s respiratory signal was acquired using a nasal cannula and was used to trigger the odor stimulation through an airflow sensor (Fig. 1B, informed consent for publication of identifying information/images in an online open-access publication have been obtain for the picture used in Fig. 1B). The air flow rate was set at 3 l/min, and the odorants were delivered over 4 s.

#### Spatio-contextual environment

Three landscape pictures presented full-screen (1280 × 1024 pixels, 72 dpi, Fig. 1B) constituted the visual contexts (a desert landscape, a coastal cliff, and a lavender field). For each of these three visual contexts, yellow circles symbolized three spatial locations, leading to a total of 9 non-overlapping spatial locations (Fig. 1A).

#### Multidimensional episodes

Three multidimensional episodes were created, each composed of three target odors (What) associated with specific locations (Where) within a given visual context (Which context). To limit associative semantic processes, the odors, spatial locations, and visual context were arbitrarily linked (the odors were not congruent with the landscapes). An in-house LabView software (version 8.6 or higher) controlled the presentation of odors, pictures, and circles and recorded the participants’ responses and breathing throughout the experiment. To interact with the software, the participants used a trackball (Kensington, Redwood Shores, CA, USA). Whenever the participants clicked on a circle, the odor stimulus was delivered at the beginning of the subsequent expiration, enabling the odor to be perceived at the beginning of the next inspiration.

### Experimental procedure

The experimental procedure consisted of four sessions performed over the course of four successive days (Fig. 1C). The first three sessions were used for encoding, and the retrieval occurred in the fourth session to assess the participants’ memory of the episodes perceived during encoding. A night of sleep at home followed each of the encoding sessions to reduce interference between episodes and to promote consolidation^45,46^. Participants had to wear a wrist actimeter (Actigraph link GT9x, Actigraph, FL, USA) on the dominant hand without interruption during the 4 days of the experiment to assess sleep parameters. While the time of the sessions differed between participants (from 9h30 to 16h30), each participant completed the four sessions at the same time of the day to limit the differential influence of internal states (e.g. hunger, digestion, fatigue) on olfactory and cognitive processes between sessions^47,48^.

#### Encoding

During encoding, participants were not given explicit instructions to memorize the episodes and they were not told that the goal of the study was to test for a possible link between dreaming and memory. They were told the following: “*In this experiment we investigate the perception of episodes comprising odors positioned at specific locations of a background picture and its influence on dream frequency*. *For 3 days*, *you will be presented each day with a new episode that you will explore for 7 minutes*. *At night you will wear a wrist actimeter measuring movements (it is to assess your sleep duration)*, *and wake up at 5am and at your usual waking time to report possible dreams*. *The 4*^*th*^ *day we will ask you about the impressions you had when you explored the episodes*.” After such instructions, participants freely discovered one episode per day for 7 min (Fig. 1C). The participants could explore the visuo-olfactory environment and smell the odors by clicking on the yellow circles in an unlimited manner (they were told to wait 20s between 2 clicks to avoid saturation of the smell sensation). The absence of memorization instruction ensured free encoding, in a similar way to what happens in real-life situations. The order of presentation of the three episodes (desert landscape, coastal cliff and lavender field) was randomized between the participants.

#### Dream recall

During each night at home following an encoding day, participants were instructed to wake up at 5 am and at their usual waking time to report dreams. 5 am was chosen rather than an earlier time in the night for the first awakening to increase the chances to get dream reports e.g.^49^. Participants were asked to record their dreams, if any, with a voice-recorder immediately after each awakening and to give all the details they could possibly recall (Fig. 1C). If they had no dream in mind, participants were asked to explicitly state it in the voice-recorder. In the morning they filled in a questionnaire about the content of the night’s dreams. For each dream, they reported (1) the amount of positive and negative emotions (1 neutral, 10 very intense), bizarreness (1 not at all, 10 completely), amount of interactions between characters, images, sounds, odors, taste and touch sensations (1 not at all, 10 many) (Q1), (2) the place(s), character(s) and actions(s), and (3) the element of their waking life that they felt were related to the dream (Q2). For each awakening, all the oneiric content reported was considered as one single dream.

#### Retrieval

Retrieval was tested on the fourth day. The Episodic Retrieval test was composed of 18 trials consisting of the presentation of 9 target and 9 distractor odors. The target and distractor odors were presented in different pseudorandom orders (no more than 3 target or 3 distractor odors in a row) for each participant.

Each trial began with an odor recognition task (Fig. 1D). The participants were presented with the odors for 4 s and determined whether they recognized the smell as having been previously presented during the encoding (“*Do you recognize this smell?*”). Two situations could happen: 1) If the participants responded “*Yes*”, they were asked to retrieve the entire episode associated with the odorant and to press on the trackball if they succeeded in less than 25 s after the odor was sent (“*Press when you remember the context*”). After this delay, they were given up to 10s to choose both the accurate visual context and the exact location of the odor by selecting one of the three pictures, and then one of the nine circles superimposed on the chosen picture. A response was considered correct when the participants selected both the accurate context and the specific location previously associated with the odor during the encoding. 2) If the participants responded “*No*”, they had to press on the trackball (“*Press the button*”) and rest until the next trial. At the end of a trial, a new odor was presented to the participant after a rest of 3s.

Following this retrieval task, the strength of the association between the spatial location and the visual context of an event was tested in the Visuo-spatial (VS) association test. The participants were presented with the three landscape pictures with all the nine circles superimposed, and they had to choose the three circles that were associated with each of the pictures during the encoding.

#### Rating of odors intensity, pleasantness and familiarity

At the end of the retrieval session, the participants were presented again with the 18 odors and were asked to rate the odorants in terms of intensity (from “extremely weak” to “extremely strong”), pleasantness (from “extremely unpleasant” to “extremely pleasant”) and familiarity (from “unknown” to “extremely familiar”) using visual analogue (non-graduated) scales. The pleasantness scale was divided into two equal parts by a “*neutral*” value separating the ratings of unpleasantness and pleasantness. The intensity, pleasantness, and familiarity ratings were transformed into scores from 0 to 10.

At the end of the experiment, the participants filled in a final questionnaire to assess: (1) how much they found the experiment difficult (on a scale from 1 to 5), (2) whether they had guessed that they would eventually be asked to retrieve the episodes perceived the 3 first days, (3) the strategy that they used to explore or memorize the episodes and to retrieve them, (4) whether they thought about the episodes between the sessions, and (5) whether they dreamt of all or parts of the multisensory episodes they discovered in the lab (Q3). If they answered yes to this last question, participants had to tell which element(s) of which episode(s) had been incorporated in which dream(s).

### Data analysis

#### Dream reports

Dreams rarely replay a complete episodic memory. Rather, they incorporate isolated elements of an episodic memory with more or less distortions^11^. As the learning phase may be replayed in a modified and partial way during dreaming, learning-related was considered as any element of a dream report resembling the specific constituent of episodes presented before the dream report, such as odors or visuo-spatial elements of the landscapes (strict scoring). The resemblance could be metaphoric as it was shown that dreams can evoke recent memories in such a way^17,50^. As done in previous studies, we also performed a more liberal scoring that included the experimental context of the learning phase (e.g. the lab’s building, experimenters, the olfactometer, the breathing apparatus also called “nasal glasses” in French).

In order to detect incorporation of the encoding phase in the dream reports of the participants, the experimenters considered the following data:

1. the audio dream report: identification of elements related to the encoding phase;
2. the waking life elements considered as linked to the dream by the dreamer in Q2;
3. the answer to the question Q3 “*In the last 3 days did you dream of all or parts of the multisensory episodes you discovered in the lab?*”.

Elements related to the episodes presented during the encoding phase were searched in the audio dream reports, in Q2, and in Q3. A dream was considered as learning-related if elements related to episodes presented before the dream report were identified in at least one of the three types of data considered. Two experimenters (JP and PR) blind to which episode was presented before each dream report realized independent scoring. When the two scorers disagreed, a consensus was reached after discussion.

#### Memory performance

Three types of memory performance were calculated. First, recognition memory of odor was assessed in the Episodic Retrieval test using parameters from the signal detection theory^51^. From the experimental conditions (target odor *vs*. distractor odor) and the participants’ behavioral responses (“*Yes*” *vs*. “*No*”), four response categories were defined: Hit and Miss occurred when the target odors were accurately recognized or incorrectly rejected, respectively, and correct rejection (CR) and false alarm (FA) occurred when the distractor odors were correctly rejected or incorrectly recognized, respectively. The odor recognition memory score (*d’*) reflecting the participant’s ability to discriminate between target and distractor odors, was calculated as follows: d’ = ln [HR (1 − FR)/FR (1 − HR)], where *HR* represents the Hit rate [(Hit + 0.5)/(*N*_*t*_ + 1)], *FR* represents the false alarm rate [(FA + 0.5)/(*N*_*d*_ + 1)] and *N*_*t*_ and *N*_*d*_ represent the number of target and distractor odors, respectively, for which the participants provided an answer. Such score may be good (positive, maximum score of 5.89) or poor (negative values, minimum score of −5.89). To assess odor recognition memory performance for each day or context of the encoding, we measured the ratio of the Hit to the number of detected target odor (Hit/detected target odor).

Second, odor-evoked episodic memory performance was calculated. In the Episodic Retrieval test, we focused the analyses on the participants’ accurate responses for the target odors (Hit). Four types of responses were defined depending on the recall accuracy. When the participants correctly recognized the target odors, they could accurately remember both the location and the context (WWW), the location only (WWhere), or the context only (WWhich) or they could be mistaken about both dimensions (What). These different scenarios were named *episodic combinations*. The theoretical proportions of these episodic combinations resulting from responses given randomly were 0.019 for WWW [1 response (“*Yes/No*”) out of 2 × 1 context out of 3 × 1 location out of 9], 0.148 for WWhich [1 response (“*Yes/No*”) out of 2 × 1 context out of 3 × 8 locations out of 9], 0.037 for WWhere [1 response (“*Yes/No*”) out of 2 × 2 contexts out of 3 × 1 location out of 9] and 0.296 for What [1 response (“*Yes/No*”) out of 2 × 2 contexts out of 3 × 8 locations out of 9]. The episodic memory score was calculated as the ratio WWW/Hit.

Third, we evaluated the visuo-spatial memory of the episodes by calculating the visuo-spatial memory score (VS). VS corresponds to the number of yellow circles accurately placed onto the three landscape images (3 yellow circles per image), and ranges from 0 to 9. The VS score evaluates the memory of the visuo-spatial location of the circles for each landscape picture presented during the encoding phase.

#### Sleep parameters

The wrist-actigraphy data were analyzed with the ActiLife software (Actigraph 2012, ActiLife 6.13.2; sampling rate 30 Hz; settings: gender, age, weight, dominant hand; Kole Kriple algorithm: minimum sleep period time = 80 min, minimum inactive time to define bed time = 5 min, minimum activity time to define waking time = 10 min) to assess two sleep parameters, the sleep period time (SPT) corresponding to the time between “lights off” and “lights on”, and the wake after sleep onset (WASO), corresponding to the period of wakefulness between the first falling asleep and the last wake up. Note that according to validation studies that compared Actigraph link GT3x performance with gold-standard polysomnography, actigraphy overestimates the average WASO^49^.

### Statistical analyses

The statistical main effects of the factors and interactions were determined using repeated measures ANOVAs followed by *post-hoc* bilateral Student *t-*tests when the main effects and/or interactions were significant. For between-groups comparisons and for the comparison with random score, we used bilateral and unilateral Student *t-*tests, respectively. The correlations between the factors were determined using the non-parametric Spearman’s rank correlation. The chi-squared test was used to determine whether distributions of categorical variables differ between groups. The effects were considered significant at p < 0.05. Effect size was determined using Cohen’s *d* for unpaired t-test. Statistical analyses were performed using Statistica (StatSoft®, Tulsa, OK, USA).

## Results

### Odor ratings

As expected from previous studies^34,35^, the odorants were considered as moderately intense (5.89 ± 1.13, on a scale of 1 to 10), moderately familiar (5.24 ± 1.10), and relatively neutral (4.81 ± 1.25).

### Dream recall

**Figure 2:**
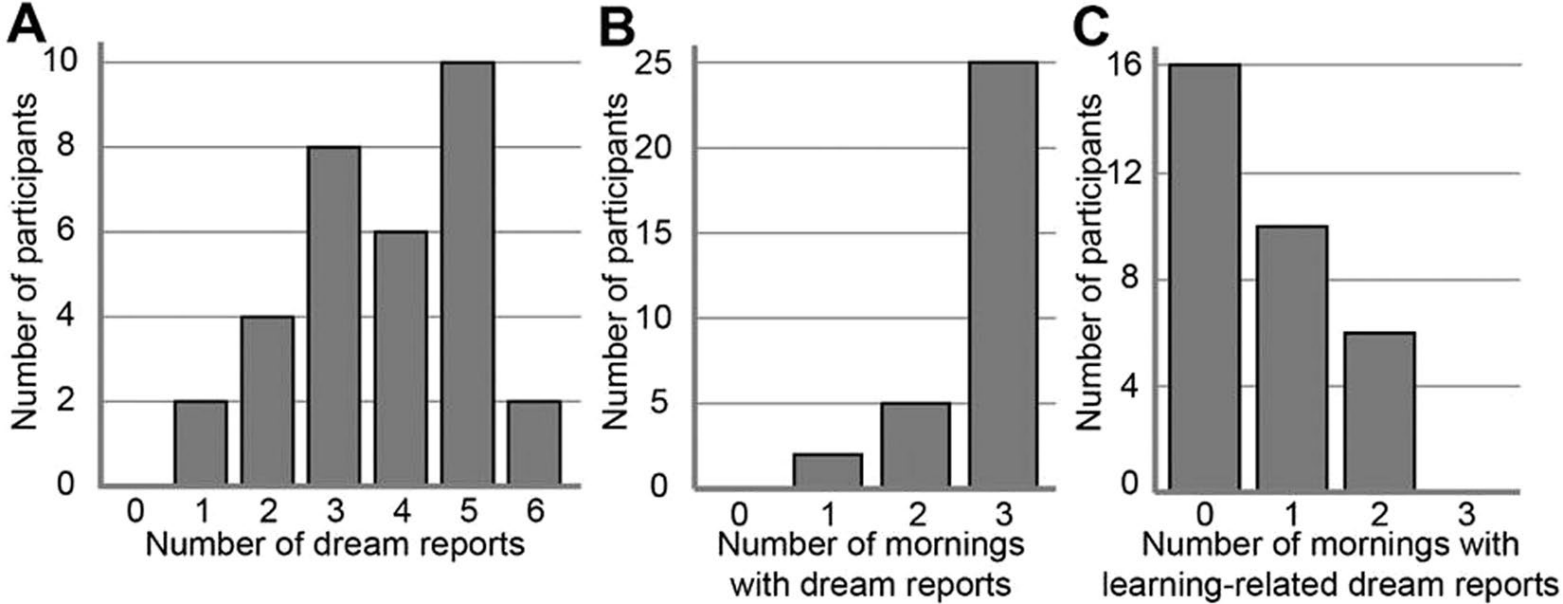
Dream reports frequency. (**A**) Number of dream reports (maximum of 6) per participant. (**B**) Number of mornings with dream reports (maximum of 3) per participant. (**C**) Number of mornings with learning-related dream reports (maximum of 3) per participant.

The 192 awakenings (32 participants × 2 awakenings per night × 3 nights) resulted in 120 dream reports (62.5%), comprising 158 ± 201 words in average (between subjects average of within subject averages = 158 ± 145 words). Of all the dream reports, 56 were reported at 5 am (actigraphy confirmed that participants awakened at 5 am, mean word count = 140 ± 209), and 64 after the awakening at the usual waking time (6.30 to 10 am, mean word count = 174 ± 193). Participants reported on average 3.75 ± 1.37 dreams (from 1 to 6) over the three nights (Fig. 2A). The majority of the participants reported at least a total of 3 dreams and at least one dream per morning (Fig. 2B).

The grand average of the oneiric parameters were as follows: clarity = 5.46 ± 1.42 (on a scale of 1 to 10), bizarreness = 5.07 ± 1.79, positive emotions = 3.32 ± 1.71, negative emotions = 3.37 ± 2.06, interactions between characters = 6.26 ± 2.37, images = 6.85 ± 1.91, sounds = 6.40 ± 2.11, odors = 1.86 ± 1.24, tastes = 1.69 ± 1.04, and touch = 3.60 ± 2.36. Further results and discussion regarding sensory perceptions in dream reports are presented in the Supplementary Material.

#### Scoring of the incorporation of learning-related elements into dream reports

The Cohen’s kappa coefficient assessing inter-rater reliability was of 0.68. The strict scoring resulted in the identification of 22 learning-related dreams reported by 16 participants (D + Learn group). On average, participants had 0.7 ± 0.8 mornings with a learning-related dream (Fig. 2C). The learning-related dreams were distributed across the first, second, and third night (n = 10/6/6, respectively). Ten participants had 1 learning-related dream, 6 participants had 2. The majority of learning-related dreams (19/22, 86%) happened the night just following the discovery of the dreamt episode, 2 learning-related dreams happened the second night after the discovery of the dreamt episode, and 1 happened the third night after the discovery of the dreamt episode. This is what could be expected according to previous results which have shown that memories from the day before the dream were more often incorporated into dreams than memories dating from 2, 3, or 4 days before the dream^11^. The average dream reports word count for dreams of the D + Learn group was 227 ± 163 and 90 ± 82 for dreams of the D-Learn group (bilateral unpaired t-test: t(30) = 3.00, p = 0.005, Cohen’s *d* = 1.06).

Learning-related elements were: odors, elements of the landscapes (cliffs, beach, sea, field of odorant herb, desert, places with arid climate), or the yellow circles. Only 5 dream reports (from 4 different participants) mentioned explicitly olfaction or odors (“I am annoyed by the cigarettes they smoke”, “a warm odor of desert”, “good odors of food”, “a fish without any odors”, “I smelt the odor of cigarette”), and only 2 oneiric odors could be related to the encoding phase. Some examples of dream reports considered as learning-related with the strict scoring are listed below.

*“I was at the top of a cliff*. *It was really resembling the one I saw in the lab yesterday”*

*“I was swimming in the sea*, *there were waves… on the beach I saw an old pair of glasses made out of metal and with curved branches to go around the ears… I scratch my nose and three boogers got out of it*, *they looked like little characters with a corn seed shape”*

*“I walk in a place where there is nothing*, *I am alone*, *I am looking for people because I’m lost”*

*“I knew that I was looking for something and that I found it*, *but I didn’t know what … it was a field of wild mint*, *it was what I was looking for”*

« *A yellow circle associated with the word lemon* »

The liberal scoring resulted in the identification of 37 learning-related dreams by 21 participants. The average word count for the dreams of the D + Learn group was 191 ± 156 and 95 ± 99 for dreams of the D-Learn group (bilateral unpaired t-test: t(30) = 1.85, p = 0.073, Cohen’s *d* = 0.73). The 15 supplementary learning-related dreams were related to the task/experiment in a metaphorical way and/or by evoking odors or taste. Some examples of dream reports considered as learning-related with the liberal scoring are listed below.

“*I dreamt that I woke up to report my dream using the voice recorder just as in the experiment*”

“*I was in the building for the study of dreams*, *downstairs in the cafeteria*, *and I was explaining to a rhinoceros that I was preparing my dreams as my hand-bags*, *with many objects which could be useful in case*”

“*The 4*^*th*^ *day I learn that some elements which could induce fear reactions were hidden in the images presented for the experiment which explained why I had rather negative dreams the last couple of days*”

“*Some guys with white coats were doing experiments on us*”

“*We had 1 hour and 30 min to drink different drinks that we did not know*, *we had to hurry*”

“*I smelt the odor of cigarette on my little brother*”

### Memory performance

#### Encoding

The participants clicked on average 18.54 ± 8.41 (range from 8.33 to 39.00) times on the circles per episode and consequently smelt 6.18 ± 2.88 times each odorant. One-way ANOVAs performed on the number of click related to each episode showed that it did not differ between days (day1, day2, day3; F(2,93) = 0.19, p = 0.83) or contexts (desert, coastal cliff or lavender field; F(2,93) = 0.15, p = 0.86).

#### Odor recognition

**Figure 3:**
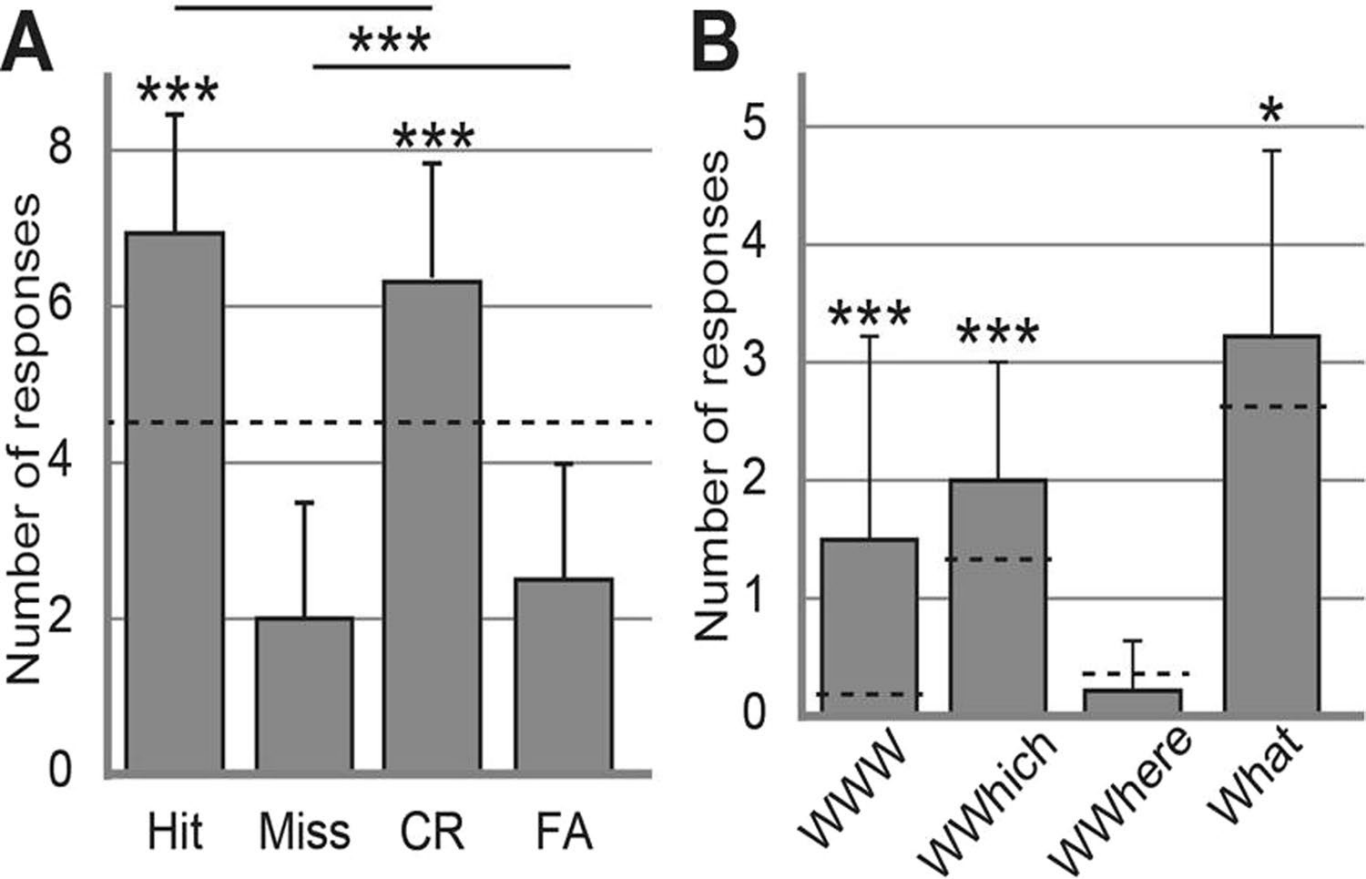
Memory performance. (**A**) Odor recognition, mean number of responses for the Hit, Miss, CR and FA responses (maximum of 9). (**B**) Episodic retrieval, mean number of responses for the WWW, WWhich, WWhere and What responses (maximum of 9). Dashed horizontal lines indicate chance levels. Vertical bars represent SD. *p = 0.05; ***p < 0.001.

The participants were presented the target and distractor odors and were asked whether they had smelled them during the encoding phase. The recognition memory score was high (*d*’ = 2.20 ± 1.35; range from −0.62 to 4.68), which indicated that the participants were proficient in recognizing target odors and rejecting distractor ones. The number of responses in the four response categories (Hit, Miss, CR and FA) is shown in Fig. 3A. The proportions of Hit (0.78 ± 0.17) and CR (0.72 ± 0.17) responses were above the chance level [0.5; unilateral t-tests: Hit, t(31) = 9.34, p = 10^−6^, CR, t(31) = 7.29, p = 10^−6^]. A two-way repeated measure ANOVA on the recognition memory responses showed that the number of correct responses (Hit + CR) was significantly higher than the number of incorrect responses (Miss + FA) [F(1,31) = 109.75, p = 10^−6^]. There was no significant interaction between Odor type (target vs. distractor) and Response accuracy (correct vs. incorrect) [F(1,31) = 3.19, p = 0.08]. The recognition memory score (d’) was not correlated with the number of clicks (Spearman’s rank correlation: r = 0.06, p = 0.76), suggesting that odor recognition performance did not depend on the exploratory behavior of the odors during the encoding. One-way ANOVAs performed on the Hit/detected target odor ratio related to each episode showed that the recognition memory performance was similar for the three contexts [F(2,93) = 0.12, p = 0.89] and for the three days [F(2,93) = 0.74, p = 0.48].

#### Episodic retrieval

When the participants recognized an odor as a target, they were asked to retrieve the spatio-contextual environment in which they smelled it. We focused our analysis on the responses following correct odor recognition (Hit). The number of responses in each categories (WWW, WWhich, WWhere, What) are presented in Fig. 3B. The proportion of complete accurate episodic response (WWW, 0.17 ± 0.19), the proportion of correct retrieval of the context but not of the location (WWhich, 0.22 ± 0.01) and the proportion of the complete inaccurate episodic responses (What, 0.36 ± 0.03) were significantly higher than what could be expected by chance [unilateral t-tests: WWW, chance = 0.019, t(31) = 4.38, p = 0.0001, WWhich, chance = 0.148, t(31) = 3.92, p = 0.0005, What, chance = 0.296, t(31) = 2.04, p = 0.05]. The proportion of the WWhere responses (correct location but incorrect context, 0.02 ± 0.00) did not significantly differ from chance level [unilateral t-test: chance = 0.037, t(31) = −1.54, p = 0.13]. On average, the episodic memory score (WWW/Hit) was of 0.20 ± 0.23 (range from 0.00 to 0.83). One-way ANOVAs performed on the episodic memory score related to each episode showed that it did not significantly depend on the visual context [F(2,93) = 0.33, p = 0.72] nor on the day [F(2,93) = 0.17, p = 0.84].

#### Visuo-spatial memory

The participants accurately recognized 7.28 ± 2.10 (out of nine, 81 ± 23%) of the spatial locations (yellow circles) associated with each visual context. This performance differed from random response [0.33; unilateral t-test, t(31) = 11.62, p = 10^−6^]. One-way ANOVAs performed on the visuo-spatial memory score related to each episode showed that it did not significantly depend on the visual context [F(2,93) = 2.53, p = 0.09] and the day [F(2,93) = 0.38, p = 0.68].

#### Memory performance as a function of dream content

According to the post-retrieval questionnaire on day 4, 14 participants had suspected a possible memory test, of which 7 thought about the episodes before Day 4 and 7 did not. By contrast, 17 participants had not suspected a memory test and thought about the episodes before Day 4. Only 1 participant had not suspected a memory test and had not thought about the episodes before Day 4.

**Figure 4:**
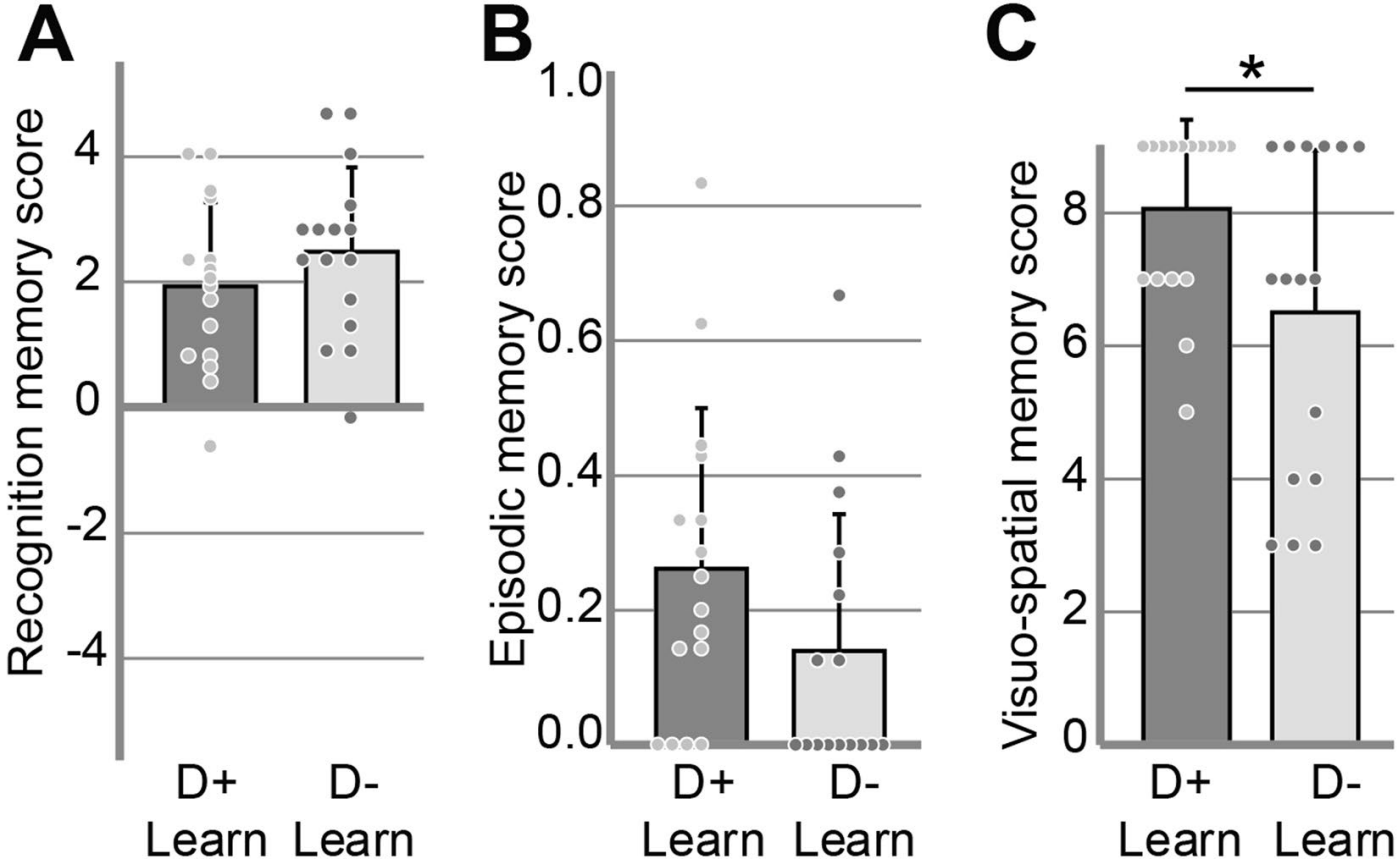
Memory performance according to the incorporation of learning-related elements into dream reports (strict scoring). Means, standard deviations and measures for each subject are represented. (**A**) Mean odor recognition memory score (d’), (**B**) mean episodic memory score (Hit/WWW) and (**C**) mean visuo-spatial memory score (VS) for the participants who had at least one identified learning-related dream report (D + Learn, n = 16) and for those with no identified learning-related dream report (D-Learn, n = 16). Vertical bars represent SD. *p < 0.05.

For the strict scoring, we compared memory performance between participants who recalled learning-related dreams (D + Learn, n = 16) and those who did not (D-Learn, n = 16). Between group comparison of the performance for odor recognition (d’) and episodic retrieval (WWW/Hit) resulted in non-significant difference (bilateral unpaired t-tests: d’ D + Learn = 1.93 ± 1.34, d’ D-Learn = 2.48 ± 1.35, t(30) = 1.15, p = 0.26, Cohen’s *d* = 0.40, Fig. 4A; WWW/hit D + Learn = 0.26 ± 0.24, WWW/hit D-Learn = 0.14 ± 0.20, t(30) = 1.57, p = 0.13, Cohen’s *d* = 0.54, Fig. 4B). However, the visuo-spatial memory score (VS) did differ significantly between the two groups. The participants who had dreams scored as learning related had a better visuo-spatial memory of the episodes than the participants who had no dream scored as learning related (bilateral unpaired t-test, VS D + Learn = 8.06 ± 1.34, VS D-Learn = 6.50 ± 2.45, t(30) = 2.24, p = 0.03, Cohen’s *d* = 0.83, Fig. 4C). The number of participants who suspected a possible memory test did not significantly differ between the D + Learn and the D-Learn groups [Chi-square test, _X_^2^(1) = 2.03, p = 0.15].

**Figure 5:**
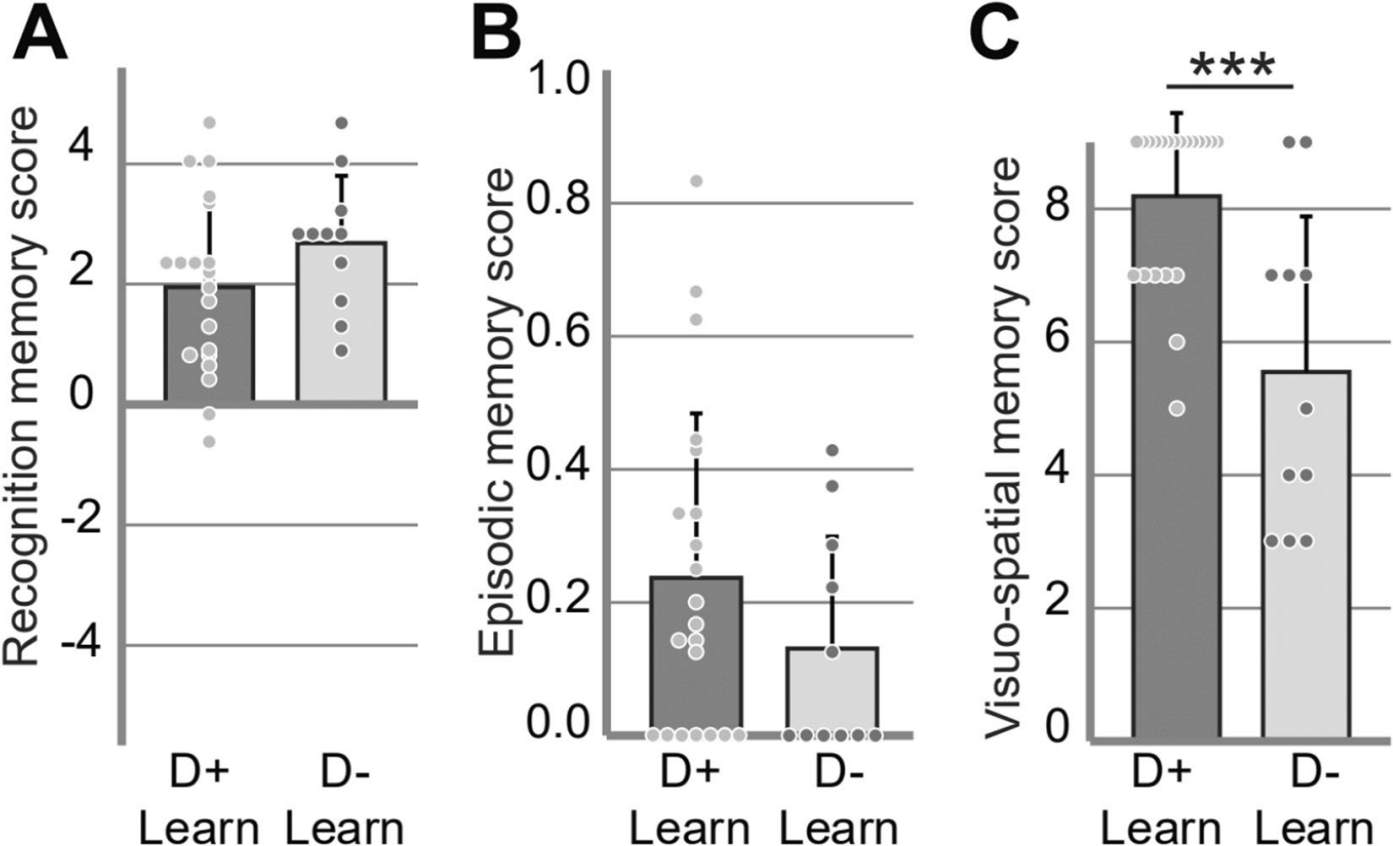
Memory performance according to the incorporation of learning-related elements into dream reports (liberal scoring). Means, standard deviations and measures for each subject are represented. (**A**) Mean odor recognition memory score (d’), (**B**) mean episodic memory score (WWW/Hit) and (**C**) mean visuo-spatial memory score (VS) for the participants who had at least one identified experiment-related dream report (D + Learn, n = 21) and for those with no identified experiment-related dream report (D-Learn, n = 11). Vertical bars represent SD. ***p < 0.001.

For the liberal scoring, we compared the memory performance between participants who recalled experiment- and/or learning-related dreams (D + Learn, n = 21) and those who did not (D-Learn, n = 11). The results (bilateral unpaired t-tests: d’ D + Learn = 1.95 ± 1.42, d’ D-Learn = 2.69 ± 1.11, t(30) = −1.48, p = 0.15, Cohen’s *d* = 0.58; WWW/Hit D + Learn = 0.24 ± 0.25, WWW/Hit D-Learn = 0.13 ± 0.17, t(30) = 1.28, p = 0.21, Cohen’s *d* = 0.51; VS D + Learn = 8.19 ± 1.25, VS D-Learn = 5.55 ± 2.34, t(30) = 4.19, p = 0.0002, Cohen’s *d* = 1.47) are presented in Fig. 5. The number of participants who suspected a possible memory test did not significantly differ between the D + Learn and the D-Learn groups [Chi-square test, _X_^2^(1) = 2.69, p = 0.10].

### Sleep parameters

We collected wrist-actigraphy data for 30 participants (2 actimeter watches did not work properly). The average sleep period time (SPT) was close to 8 hours (486 ± 49 min) and the average wake after sleep onset (WASO) was 73 ± 24 min. Memory performance was not significantly associated with the SPT or WASO (Spearman’s rank correlations: WASO & *d*’: r = 0.30, p = 0.11; WASO & WWW/Hit: r = 0.22, p = 0.25; WASO & VS: r = 0.20, p = 0.29; SPT & *d*’: r = −0.08, p = 0.64; SPT & WWW/Hit: r = 0.21, p = 0.27; SPT & VS: r = 0.32, p = 0.09).

## Discussion

The objective of this study was to provide further data to test whether recalling a dream related to a recent experience was associated with improved post-sleep memory of this experience. The protocol used has several benefits compared to previous ones. First, it increases the chances of obtaining numerous dream reports (2 awakenings per night during 3 nights in high dream recallers using a voice recorder), and thus the chances to get learning-related dream reports, while keeping sleep disruption to a minimum (at-home recording with only one induced awakening per night). Second, it introduces a sensory perception rarely explored – olfaction – which is a non-dominant sense in wake and dreams (at least in modern industrialized countries). Third, by using pictures with specific, highly distinguishable attributes which are rare in the participant’s daily life (landscapes of coast cliff, desert, and lavender field, Fig. 1A), the specific detection of learning-related elements into dream reports is more likely. Finally, this protocol is closer to real-life situations than previous paradigms due to the use of non-explicit memory encoding (i.e. each unique episode is experienced shortly with no explicit instructions to memorize it), which better fits with the content-based definition of episodic memory^52,53^. The use of such a paradigm resulted, as expected, in an important amount of learning-related dream reports (22 from 16 subjects for the strict scoring and 37 from 21 subjects for the liberal scoring). The elements of the task incorporated into dreams were most often elements of the landscape pictures, but rarely odors. The participants who reported learning-related dreams (and those who reported learning-related and/or experiment-related dreams) did not have significantly better performance at odor recognition nor odor-evoked episodic memory. However, they did have better visuo-spatial memory of the episodes than other participants. These results suggest a link between dream content and memory consolidation and support the hypothesis of the learning phase being loosely incorporated into dreams and this incorporation being associated with sleep related memory consolidation.

### Sleep and memory performance

According to the wrist-actigraphy data, the participants slept on average approximately eight hours, a result that is consistent with a recent epidemiological survey about the sleep and dream habits of healthy Lyon University students^39^. The average waking time after sleep onset (WASO) was approximately 75 min, which is more than what can be expected in young and healthy high dream recallers^37^. This high amount of WASO is partly explained by the 5 am awakening but is also most probably overestimated. Validation studies have indeed shown that in comparison to gold-standard polysomnography, wrist actigraphy overestimates WASO^54^. In addition, the participants wore the actimeter watch on the dominant hand whereas it is usually worn on the non-dominant hand which may have in turn increased the number of movements, resulting in the false positive for wakefulness detection. Importantly, no significant correlation between the sleep parameters and memory performance was observed.

Odor recognition and episodic retrieval performance followed a pattern similar to that reported by Saive *et al*.^35^. In other words, if the dream report part of the protocol might have induced some stress in participants (e.g. some of them may have feared not to succeed in reporting dreams), it did not seem to have affected memory performance. Similarly, the single nocturnal awakening apparently did not affect the sleep-related memory consolidation process, which is coherent with previous results showing no alteration of memory performance after few awakenings per night^21,55^. Altogether, the results suggest that the experimental paradigm did not disturb the memory consolidation process known to be at play during sleep^45,46^.

### Dream reports

An important number of dream reports were obtained by recruiting high dream recallers and asking them to awaken two times per night (i.e. only one middle sleep awakening) during the three days of the experiment, once at 5 am and once at their usual waking time. The great majority of the participants reported at least three dreams (average number of dreams reported = 3.7 ± 1.4).

The paradigm of Saive *et al*.^34,35^ made it possible to specifically and accurately identify learning-related elements within dream reports, as demonstrated by the good between-scorers agreement. The scoring of dream reports for incorporation of the encoding phase resulted in the detection of an important number of learning-related dreams (18% for the strict scoring with n_dreams_ = 22 and n_subjects_ = 16, 31% for the liberal scoring with n_dreams_ = 37 and n_subjects_ = 21). The protocol thus succeeded in yielding an important number of learning-related dream reports, which in turn enabled a between-group comparison of memory performance according to dream report content in a satisfying (statistically speaking) number of participants.

Even if odors are rarely explicitly mentioned in dream reports^56^, we expected that presenting unusual odors the day before the night of dream collection would increase the chances of their incorporation into dreams. We indeed know from previous work that among all the memories incorporated into dreams a large part date from the day before (the so-called “*day residues*”)^11^. This strategy resulted in only a weak increase (at the descriptive level) in the percentage of dream reports with explicit mention of odors (e.g. 4% in our study vs 1% in Zadra *et al*.^53^), coherently with previous results^57^. In other words, our protocol did not succeed in improving greatly and significantly the amount of incorporation of odors into dream reports. The incorporation rate of odors into dream reports seems to mostly depend on olfactory expertise^58,59^.

### Memory performance as a function of dream report content

The between-group comparisons of memory performance yielded significant effect for the visuo-spatial memory of the episodes, be it for the strict or the liberal scoring (Figs 4 and 5). The participants who had dreams scored as learning-related had a better visuo-spatial memory of the episodes than the participants who had no dream scored as learning-related. Moreover, participants who dreamt of the cliff episodes (the most dreamt episode) tended to show a better visuo-spatial memory of this episode than the other participants (see the Supplementary Data). This result is in favor of a link between memory consolidation and learning-related dreams.

One tricky issue about testing a possible effect of incorporating a task into dreams on memory performance is where to draw the limit between elements related to the task versus not related to the task. In this study, elements specific to the learnt episodes in dream reports were initially identified (strict scoring). Then, a second, more liberal scoring that also included elements that were specific to the context of the experiment, such as the lab building, the experimenters, and the experimental setup, was carried out. This second method resulted in more learning-related dreams and interestingly yielded the same results as the conservative scoring but with an even larger main effect of the improved visuo-spatial memory of the episodes in participants who had learning-related and/or experiment-related dreams (Fig. 5). This result suggests that the incorporation into dreams of elements related to the task and the incorporation into dreams of elements related to the context of the encoding phase, can both be considered as associated with the memory consolidation process taking place during night sleep.

It is unclear why participants with learning-related dreams did not show a better olfactory memory than participants with no learning-related dream reports. There are several potential explanations for this result. First, it is possible that only the memory elements that are incorporated into dreams are better consolidated. Consistent with this, participants of the D + Learn group dreamt predominantly of the landscape pictures and only little about the odors, and had better scores than the D-Learn group only for the visuo-spatial memory. Second, it could be that when incorporated into a dream, elements of the episodes as well as elements of the experimental context trigger the reactivation of the memory trace formed during the encoding phase (because the dreaming brain is fundamentally associative^50,60^), as would do a context-related stimulation presented during sleep^61–63^. In this experiment the improved performance in the visuo-spatial domain but not in the olfactory domain for the D + Learn group could be explained by the fact that the visuospatial representation of the episodes was more easily reactivated during sleep than the olfactory representation, whatever the element triggering the memory trace was (visual or olfactory). It could be due to the lack of olfactory expertise of the participants, i.e. to an insufficient perceptual sensitivity and imagery ability in non experts^64^, or to the unfamiliarity of the odors presented (they were difficult to verbalize and to associate to known odors), or to a combination of these factors. Finally, given the few studies that have tested the impact of sleep on olfactory memory consolidation, one cannot exclude that sleep is less critical for olfactory memory consolidation than for the consolidation of memories in other sensory modalities. This would be coherent with the fact that olfaction and taste are typically the less-represented sensory modalities in dream reports (see Supplementary Data).

As a whole, our findings suggest (1) that the incorporation of an element of the task into dreams is associated with the reactivation during sleep of the encoding phase’s memory trace, yielding improved memory performance and, (2) that during sleep in our study, the olfactory component of the memory trace was less reactivated than the visuo-spatial component.

Regarding the possible interaction between sleep stages and the effect of interest (improved memory performance when dreams incorporate a recent learning), the present study do not provide helping data because the sleep stages at awakening were not recorded. It would be worth testing in future studies whether NREM sleep learning-related dreams show more often a link with improved memory performance than REM sleep learning-related dreams and whether this possible effect is dependant on the task used.

### Limitations and theoretical considerations

To interpret the results and to discuss the possible answers to the original question asked in the introduction of this article, one has to bear in mind the theoretical and methodological considerations below.

First, given that longer dream reports were observed in the D+Learn group as compared to the D-Learn group for the strict scoring, one cannot exclude that the more important amount of learning-related dream reports in the D + Learn group is due to a better sampling of the dreaming activity in this group. As visual memory has been related to dream recall frequency and dream length in some studies^65^, the difference in word count between the D + Learn and D-Learn groups could explain why the D + Learn group improved significantly more on the visual component of the task only. However, given that dream length did not significantly differ between the two groups for the liberal scoring, this hypothesis does not seem the strongest, but would be worth testing more thoroughly in future studies.

Second, the occurrences of the learning-related elements in the dreams reported before the experiment were not measured, and it is thus difficult to know whether those elements were incorporated into dream reports during the experiment on a level that exceeds the chance level. However, for 86% of the learning-related dreams, the episode incorporated was discovered by the participants the day before, which shows that the incorporation of the episodes into dreams was not randomly distributed across nights. Rather, it was biased towards what would be expected according to the known rules of incorporation of recent memories into dreams (more incorporation of episodes discovered the day before than of episodes discovered 2 and 3 days before the dream^11^).

Third, learning-related dream reports may act as a reminder of the task and induce a cerebral reprocessing of the encoding phase. This effect may contribute to the improved performance observed in the D + Learn group.

Fourth, given that dream content is often social e.g.^66^, it may be pertinent to use a memory task with a social dimension to test a possible link between incorporation of memories into dream content and memory consolidation in future studies.

Fifth, in the present study, the inter-scorer agreement for the scoring of dreams as learning-related was moderate (kappa = 0.68). It can probably be explained by the fact that we considered both strict and loose associations with the learning phase to score dream content as learning-related. For the loose associations, a great extent of possible fragmented and metaphoric representations of the task in the dream content was possible, which made the scoring much more difficult and subjective than when only strict associations are considered. Even if moderate, a kappa of 0.68 may be thus considered as quite convincing, given the difficulty of scoring loose associations.

More generally, while testing whether the incorporation of a learning-phase into dream reports is associated with improved memory performance is feasible (Table 1), it is not what dream scientists truly want to test. What dream scientists are really interested in is whether the incorporation of a learning-phase into *dreams* (not dream *reports*) yields better memory performance. The problem is that we cannot test this last hypothesis, because we cannot access all the dreams someone had during his/her night of sleep. Repeatedly awakening the dreamer to ask for dream reports might increase the sampling of the dreaming activity, but this could also lead to a serious alteration of sleep and to undesired impairments of the normal memory consolidation process^30,31^ (a limited amount of awakenings per night may not disturb declarative memory consolidation^21^, but repetitive and numerous awakenings per night may severely impair memory consolidation^30–33^). And even in the unlikely event that we could have a dream report for all the dreams of the night, there is still the issue that dream reports are a posteriori subjective reports, and therefore do not equal the dream experience (reports may be partial and distorted by the waking brain). As a consequence, for all the experiments presented in Table 1, we do not know, and cannot know for a fact whether those who had no learning-related dream reports did not dream of the learning phase at some unknown point during their sleep. If some did, the results are biased because these participants have been put in the no learning-related dream group whereas they should de facto have been placed in the learning-related dream group. This problem may explain the inconsistent results obtained so far in the literature, even with the same paradigm used several times by the same team (Table 1). In the present study, our hypothesis was that the participants who have more experiment- or learning-related dream reports do dream more of the learning phase than those who do not report experiment- or learning-related dreams (if learning-related dreams happen regularly during sleep and if participants are randomly awaken during sleep to get dream reports, one should get more learning-related dream reports in those who dream more of the learning phase). In this case, and only if this assumption is true, our results would argue in favor of a link between sleep related memory consolidation and dreaming of recently formed memories.

As mentioned earlier, another complex and critical issue relates to the method of scoring dream reports as learning-related or not. We know from our own experience and from previous work that dreams nearly never reproduce an episodic memory^67,68^, but are rather composed of elements more or less transformed from several memory traces e.g.^11^. We also know that our brain does function in an associative way^17,50^, and even more so during sleep (during which the dorsolateral prefrontal cortex is hypo active^50,60,69^). Therefore, there are strong reasons to believe that if a learning phase is incorporated into dreams, it should be partial and possibly transformed. The tricky issue is then to draw a line, that is, do we only score as learning-related the dream reports that explicitly and precisely mention an element of the learning phase (only the task/stimuli to learn or also the context), or should we also consider modified/transformed/associated elements, and if so, using which criterion? The question remains open, and the different methods used to score dreams as learning-related in previous studies may explain inconsistencies in the results (Table 1). Regarding the context, previous results have shown that when stimuli (sounds or odors) representing the context of the learning phase were presented during sleep, it reactivated the cerebral representation of the memory trace and led to improved memory performance the next day^61–63^. The results of the present study showed that when the context of the experiment is considered as learning related, the memory improvement is even larger than when only specific incorporations are considered. These results speak for the consideration of the context in dreams as learning-related. Regarding transformed memories of the learing phase or memories associated with the learning phase, further studies are needed to decipher how to recognize them in dream reports and how to score them (as learning-related or not).

## Supplementary information

Supplementary information
