# Supplementary material for: Incorporation of fragmented visuo-olfactory episodic memory into dreams and its association with memory performance
Source: Sci Rep. 2019 Oct 30;9:15687. doi: 10.1038/s41598-019-51497-y (PMC6821835; doi:10.1038/s41598-019-51497-y)
Supplement: Supplementary file 1 — Supplementary information [file 41598_2019_51497_MOESM1_ESM.pdf]

Supplementary data

Supplementary figures and discussion about the sensory perceptions in dreams

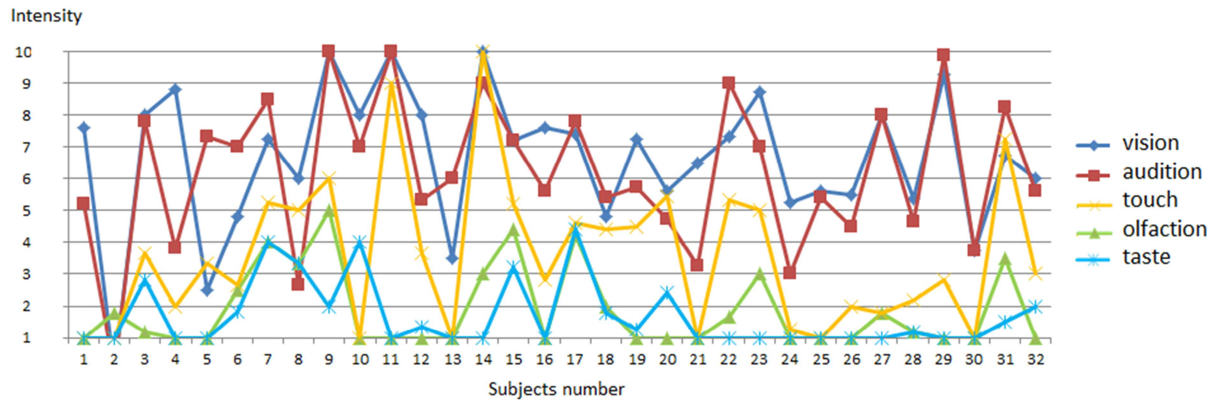

Supp Figure 1. Within subjects average of vision, audition, touch, olfaction and taste intensity in dream reports.

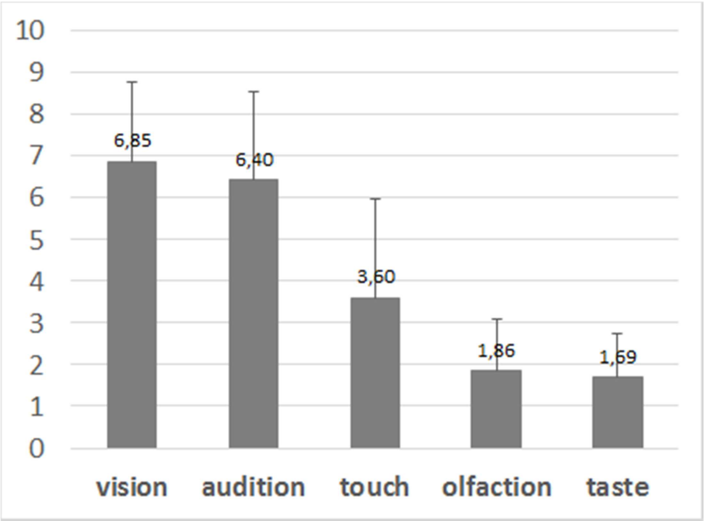

Supp Figure 2. Between subjects average (± standard deviation) of within subjects average of vision, audition, touch, olfaction and taste intensity in dream reports.

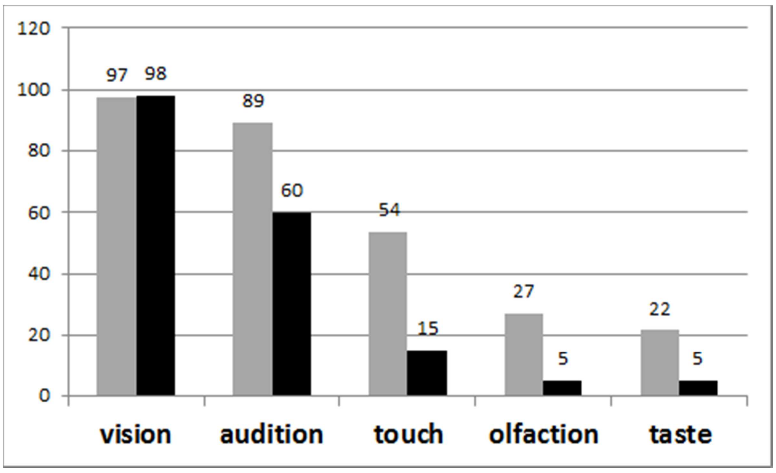

Supp Figure 3. Percentage of all dream reports (N=120) with intensity score superior to 1 for vision, audition, touch, olfaction and taste (grey). For comparison, are represented in black the percentages of dreams with explicit mention of vision, audition, touch, olfaction and taste reported in Schwartz & Maquet (2002).

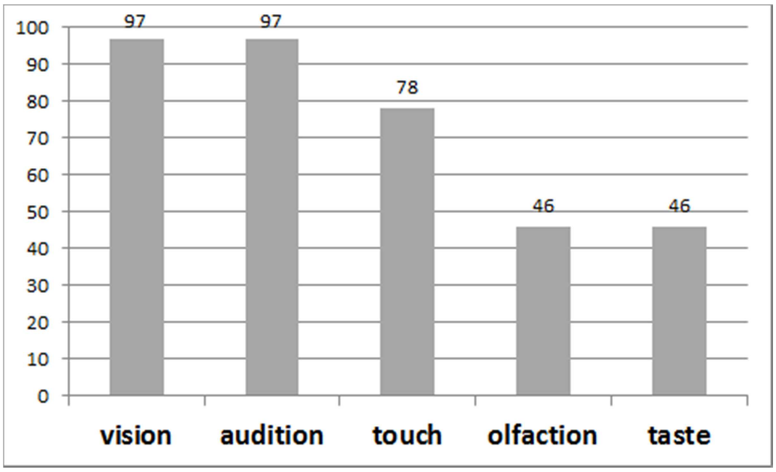

Supp Figure 4. Percentage of participants (N=32) with an average sensory intensity in dream reports superior to 1 for vision, audition, touch, olfaction and taste.

The general predominance of vision and audition over olfaction and taste in dream reports appears clearly. Touch is represented at an intermediate level between vison and audition on the one hand, and olfaction and taste on the other hand. Importantly, compared to the spontaneous mention of sensory perceptions in dream reports (Zadra et al. 1998, Schwartz & Maquet 2002), the use of systematic scales to rate oneiric sensory perceptions resulted in a global increase of the representation of the rarely explicitly reported perceptions (taste, olfaction, touch) and also of audition. Indeed, the average intensity scores (within subjects average, averaged between subjects) between vision and audition were not significantly different (vision = 6.85 ± 1.91, audition = 6.40 ± 2.11) and 31/32 participants (97%) had an average score > 1 for vision and audition (average score for all the dreams reported during the experiment). These results suggest an as frequent and as intense representation of vision and audition in the dreams of young healthy subjects. The average intensity score for olfaction was 1.86 ± 1.24 and 15/32 participants (46%) had an average score > 1

for olfaction. This is much more than what was observed by Zadra et al. (1998) who reported explicit mention of olfaction in dreams in only 15% of the participants who kept a dream diary for 2 or 3 weeks (N=164). The results are similar for taste, the average intensity score was 1.69 ± 1.04 and 15/32 participants (46%) had an average score > 1 for taste. This is also more than what was observed by Zadra et al. (1998) who reported explicit gustatory dreams in only 13% of the participants. Finally, for touch the average grade was 3.6 ± 2.36, 25/32 participants (78%) had an average score > 1 and 54% of all dream reports had a score superior to 1. This is also more than 15% of dream reports with an explicit mention of tactile sensations reported in Schwartz & Maquet (2002).

Supplementary results about the effect of the specific content dreamt

For the strict scoring, among all the learning-related dreams:
12/22 of the learning related dreams were related to the Cliff/Sea
6/22 of the learning related dreams were related to the Desert
4/22 of the learning related dreams were related to the Lavender field

To test whether a specific improvement in the episodes that were dreamt of was observed, we compared the performance for the Cliff episode between the participants who dreamt of a cliff (D+Cliff, n = 12) and the others (D-Cliff, n = 20). The performance at the Cliff episode did not differ significantly between the D+Cliff group and the D-Cliff group but a tendency was observed for the visuo-spatial memory score (bilateral unpaired t-test: hit/target D+Cliff = 0.69 ± 0.36, hit/target D-Cliff = 0.80 ± 0.23, t(30) = -1.02, p = 0.31, Cohen's *d* = 0.36; EM D+Cliff = 0.22 ± 0.29, EM D-Cliff = 0.21 ± 0.32, t(30) = 0.06, p = 0.94, Cohen's *d* = 0.03; VS D+Cliff = 2.67 ± 0.65 , VS D-Cliff = 2.10 ± 0.97, t(30) = 1.79, p = 0.08, Cohen's *d* = 0.69). EM, episodic memory score. VS, visuo-spatial memory score.
